# miR-21, miR-221, miR-29 and miR-34 are distinguishable molecular features of a metabolically unhealthy phenotype in young adults

**DOI:** 10.1371/journal.pone.0300420

**Published:** 2024-04-25

**Authors:** Alejandro Méndez-Mancilla, Eneida Turiján-Espinoza, Mariela Vega-Cárdenas, Gloria Estela Hernández-Hernández, Edith Elena Uresti-Rivera, Juan M. Vargas-Morales, Diana P. Portales-Pérez

**Affiliations:** 1 Laboratory of Immunology and Cellular and Molecular Biology, Faculty of Chemical Sciences, Autonomous University of San Luis Potosí, San Luis Potosí, San Luis Potosi, Mexico; 2 Translational and Molecular Medicine Department, Research Center for Health Sciences and Biomedicine (CICSaB), Autonomous University of San Luis Potosí, San Luis Potosí, San Luis Potosi, Mexico; 3 Laboratory of Clinical Analysis, Faculty of Chemical Sciences, Autonomous University of San Luis Potosí, San Luis Potosí, San Luis Potosi, Mexico; Federal University of Minas Gerais: Universidade Federal de Minas Gerais, BRAZIL

## Abstract

Discrepancies between the measurement of body mass index (BMI) and metabolic health status have been described for the onset of metabolic diseases. Studying novel biomarkers, some of which are associated with metabolic syndrome, can help us to understand the differences between metabolic health (MetH) and BMI. A group of 1469 young adults with pre-specified anthropometric and blood biochemical parameters were selected. Of these, 80 subjects were included in the downstream analysis that considered their BMI and MetH parameters for selection as follows: norm weight metabolically healthy (MHNW) or metabolically unhealthy (MUNW); overweight/obese metabolically healthy (MHOW) or metabolically unhealthy (MUOW). Our results showed for the first time the differences when the MetH status and the BMI are considered as global MetH statures. First, all the evaluated miRNAs presented a higher expression in the metabolically unhealthy group than the metabolically healthy group. The higher levels of leptin, IL-1b, IL-8, IL-17A, miR-221, miR-21, and miR-29 are directly associated with metabolic unhealthy and OW/OB phenotypes (MUOW group). In contrast, high levels of miR34 were detected only in the MUNW group. We found differences in the SIRT1-PGC1α pathway with increased levels of SIRT1+ cells and diminished mRNA levels of PGCa in the metabolically unhealthy compared to metabolically healthy subjects. Our results demonstrate that even when metabolic diseases are not apparent in young adult populations, MetH and BMI have a distinguishable phenotype print that signals the potential to develop major metabolic diseases.

## Introduction

Obesity is a public health concern and is directly associated with several diseases, including type II diabetes (T2D), cardiovascular and neurological diseases, and different types of cancer [1, 2]. A total of 19.2 million Mexican adults are reportedly affected by obesity according to their body mass index (BMI) and concurrently have at least one component of Metabolic Syndrome (MetS) [3]. The dysregulations collectively known as metabolic syndrome include hyperglycemia, hypertriglyceridemia, hypo-high density lipoprotein (HDL), cholesterolemia, hypertension, and a pro-inflammatory state [5–7]. Higher values of BMI have shown a progressive increase in cardio-metabolic risk [4]. However, the BMI by itself does not distinguish lean and fat tissues, which explains previous observations that a significant proportion of overweight (~50%) and obese (~30%) individuals do not show any metabolic alterations or health complications [5, 6]. Of the components of MetS, HDL-cholesterol [7] and triglycerides (TG) [8, 9] levels have greater predictive power for classifying metabolic health over waist circumference (WC), systolic and diastolic blood pressure (SBP/DBP), and glucose. Therefore, there is a need for a more accurate assessment of metabolic health at the individual level and the categorization of individuals beyond their BMI.

Metabolically healthy obesity (MHOW) implies the absence of metabolic disturbances [10–12] in individuals with a BMI above 30 kg/m^2^ [13]. MHOW individuals have a reduced risk profile of developing metabolic diseases, such as type 2 diabetes mellitus (T2D) and cardiovascular disease (CVD), and show lower mortality than metabolically unhealthy overweight/obese individuals (MUOW) [14]. Certain studies have demonstrated that the classification of the MHOW phenotype represents a transient state that can progress to a MUOW phenotype in about one-third of MHOW [15]. Although these concepts are currently introduced to explain some inconsistencies in human studies, the underlying causes are poorly understood [16]. Therefore, there are no well-established and universally accepted MHOW and MUW classification criteria.

On the other hand, retrospective studies have shown that weight gain is more rapid from young adulthood during this period, and excess adipose tissue tends to accumulate predominantly in this phase of life, with higher odds of developing CVD risk factors in adulthood [17, 18]. Hence, a better understanding of the onset of obesity and its complications during the early stages of life may help uncover novel targets and key factors to prevent the transition to metabolic diseases [19–21]. The molecular mechanisms that control the onset and progression of metabolic or non-communicable diseases (NCD) and explain the inconsistent association of BMI and metabolic health are poorly understood. Thus, it is relevant to research markers of inflammation, adiposity, and epigenetics in MUNW, MHOW, MUOW, and MHNW (metabolically healthy normoweight) individuals to explain this condition.

Several studies have proposed that members of the sirtuin (SIRT) family are metabolic regulatory sensors because they play a key role in modulating and maintaining energy metabolism and coordinating cellular responses [17]. Sirtuin 1 (SIRT 1) is the most extensively studied member and is involved in the activation of PPAR-α in response to deacetylation of PGC-1α, leading to increased oxidation of fatty acids [22–24], among other physiological functions.

Besides inflammatory cytokines, adipokines are master regulators of metabolic effects such as insulin resistance, inflammation, and cardiovascular function, causing NCD [25, 26]. Thus, it has been widely employed to describe the health status associated with BMI since these bioactive proteins are biomarkers easily detectable in the circulation, describing the general metabolic/inflammatory status. The best-described adipokines and cytokines associated with inflammation and BMI are adiponectin, leptin, ghrelin, c-peptide, and resistin. In the case of adipokines, the major circulating cytokines IL-1β, IL-8, IL-10, and IL-17A represent suitable biomarkers of inflammatory status, among others (Peptide YY and FGF-23) [11, 27]. Describing both biomarkers provides a comprehensive way of determining the status of the immunometabolism interphase since the dysregulation of these biomarkers may contribute to the development of metabolic complications.

Circulating microRNAs (miRNAs) are related not only to metabolic health and BMI but also to adipokine and cytokine expression targeting critical genes associated with immunometabolism: miR-21 (PTEN, FOXO1, HIF1α), miR-29a (PGC-1α, CEBPD, PPARα), miR-34a (PTEN, SIRT1, KLF4, ADIPOR2) and miR-221(ADIPOQ, ADIPOR1, DDIT4). These may be relevant to metabolic regulation since they are closely related to the regulation of fat deposition and modulation of the expression of signaling molecules in inflammation, metabolism, signal transduction, and cell proliferation in metabolic tissues [28–30]. The relationship between circulating miRNAs and the main components of MetS in the context of obesity in adults aged 18–70 years has been reported [31]. However, no research has specifically proposed deciphering the main epigenetic, adiposity, and inflammatory markers that confer a distinctive marker among phenotypes of metabolic health in late adolescence.

Given these knowledge gaps, we aimed to categorize the healthy and unhealthy young adult population and perform detailed metabolic phenotyping, including the molecular marker signature (cardiometabolic, adiposity, inflammation, and epigenetic markers) in the blood to provide a better explanation of the function of adipose tissue, its effects at the systemic level and the prevention of obesity. In the present study, we hypothesize that metabolic health status shows a distinctive molecular signature (circulating miRNA expression and metabolic/inflammation biomarkers) that is not observed when considering BMI alone as the selection criterion and will allow better differentiation of the phenotypes of metabolic health.

## Materials and methods

### Study population

A cross-sectional study was conducted on 1469 young adults aged 18–24 years who were applicants to a public university in Mexico recruited by the Health Services Department during the standard admission process in July 2016. The health screen consisted of 1) anthropometric measurements, including height and weight; 2) a blood draw following an overnight fast for biological markers; and 3) blood pressure measurement. Students with a previous diagnosis of diabetes, cardiovascular, autoimmune, or renal disease or cancer and those under treatment with anti-inflammatories and steroids at the time of recruitment were excluded. The Ethics Committee approved this study from the Faculty of Chemical Sciences, part of the Autonomous University of San Luis Potosí (CEEID-FCQ- CEID2015060). Written informed consent was received from all participants. All the questionnaires and data were collected anonymously, and no personal identifying information was collected.

### Stratification according to BMI and metabolic health status

The sample size was determined in a population of 1117 individuals based on the selection criteria with complete anthropometric, clinical, and biochemical profiles considering the Norma Oficial Mexicana *NOM-037-SSA2-2010* classification outlines and the American Heart Association [12]. Anthropometric measurements were performed by trained personnel; systolic blood pressure (SBP) and diastolic blood pressure (DBP) were measured on the dominant arm in the sitting position following clinical standards. The BMI classification was according to the World Health Organization (WHO), and two categories were established: 1) normal weight (BMI 18.5–24.9 kg/m^2^) and 2) overweight/obesity (BMI 25–39.9 kg/m^2^).

Serum glucose (mg/dL), triglycerides (mg/dL), total cholesterol (C) (mg/dL), HDL-C (mg/dL), LDL-C (mg/dL), uric acid (mg/dL) and insulin (mU/L) were determined by standard methods at the Clinical Research Laboratory of the Chemistry School at the UASLP in Mexico.

The groups were classified by biochemical and anthropometric parameters, considering the body mass index (BMI) and cut-off points for all cardiometabolic risk factors according to the NCEP ATP III criteria (triglycerides and HDL-cholesterol).

Then, metabolic health was stratified into four subgroups: Metabolically Healthy Normal Weight (MHNW), triglyceride level <150 mg/dL, and HDL-C level >40 mg/dL.

- Metabolically Unhealthy Normal Weight (MUNW), triglyceride level >150 mg/dL and HDL-C level <40 mg/dL.
- Metabolically Healthy Overweight/Obese (MHOW), triglyceride level <150 mg/dL and HDL-C level >40 mg/dL.
- Metabolically Unhealthy Overweight/Obese (MUOW), triglyceride level >150 mg/dL and HDL-C level <40 mg/dL.

**Fig 1:**
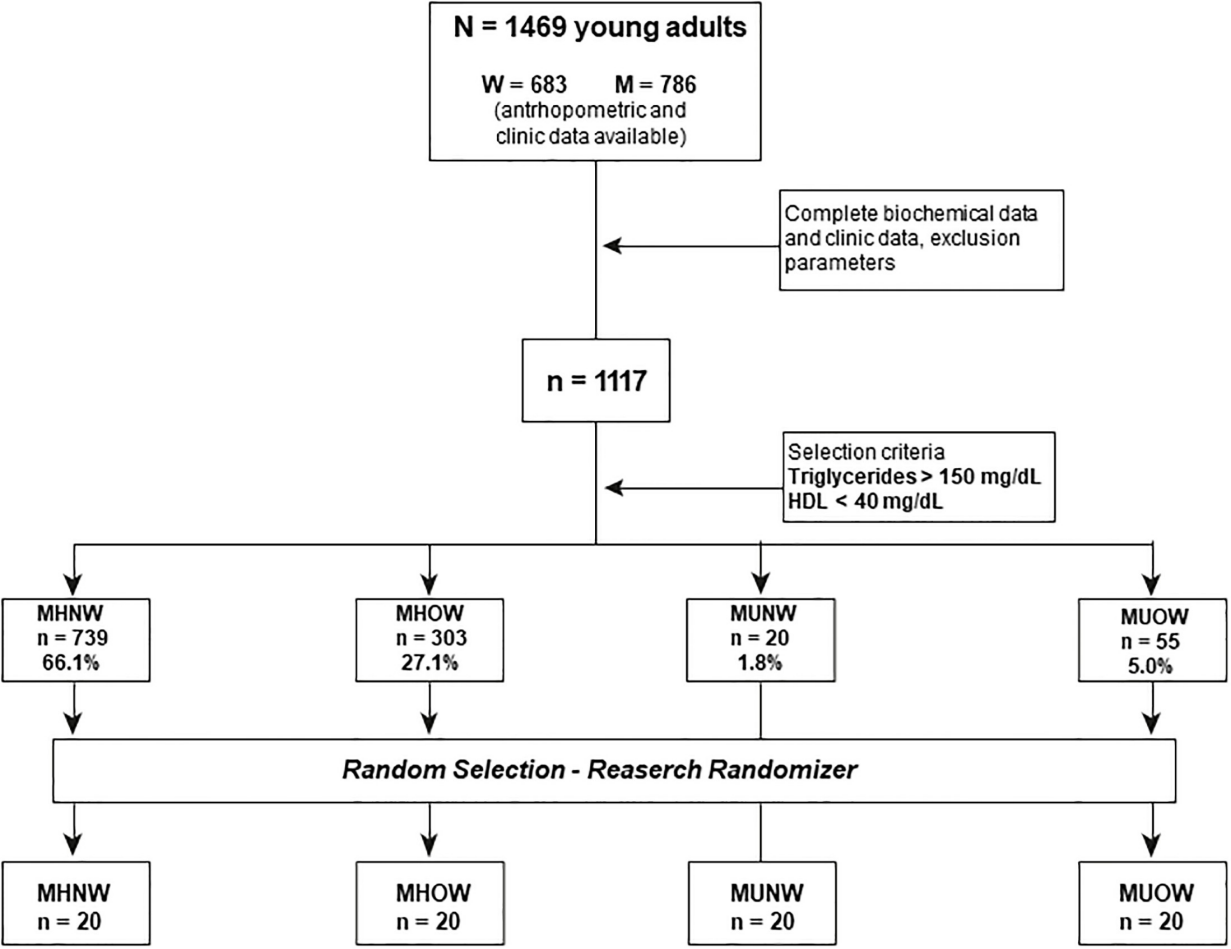
Overall study design flowchart.

Therefore, 80 individuals were randomly selected using the Research Randomizer (Version 4.0). The design of the study is illustrated in Fig 1.

### Blood samples and processing

Peripheral blood samples were collected from all participants under fasting conditions. The plasma, serum, and packed globular cell samples were centrifuged and stored at −80°C until use. Clinical data were collected from the Health Services Department. Biochemical parameters were measured and collected in the Laboratory of Clinical Analysis, Faculty of Chemical Sciences, Autonomous University of San Luis Potosí.

### Circulating miRNA isolation and qRT-PCR

Total RNA was isolated from 250 μL of plasma using TRIzol^®^ reagent (Invitrogen, USA), following the manufacturer’s instructions, with minor modifications. The quantity and purity of RNA were determined using a Synergy^™^ HTX spectrofluorometer (Biotek Instruments). A 100-ng amount of each RNA sample was used to synthesize cDNA with the Taqman^®^ MicroRNA Reverse Transcription Kit (Applied Biosystems, USA); custom RT primers for hsa-miR-21, hsa-miR-34a and hsa-miR-221 (S3 Table) and Applied Biosystems miRNA Taqman Assays for hsa-miR-29a (Assay ID #002112). 200 ng of cDNA was used to amplify miRNA by real-time PCR (qPCR) with custom primers and using the iTaq Universal Probes Supermix (Bio-Rad, USA). All qPCR reactions were duplicated in a CFX96 Touch Thermocycler (Bio-Rad, USA). A spike-in control cel-miR-39 was used to normalize expression data. Relative miRNA expression was calculated using the 2-ΔΔCt method.

### Cytokine and adipokine levels

Cytokine and adipokine concentrations in serum samples were measured with Bio-Plex Pro^™^ Human Cytokine and Diabetes custom Assays (Bio-Rad, USA), simultaneously detecting interleukin-1β (IL-1β), interleukin-8 (IL-8), interleukin-10 (IL-10), interleukin-17 (IL-17), peptide-C, leptin, resistin, ghrelin and FGF-23 and peptide YY in a single serum sample. All determinations were measured in duplicate and performed according to the manufacturer’s instructions. The data were acquired using the MAGPIX-EXPONENT^®^ system and using xPONENT^®^ Software v3 (Luminex Corp). The adiponectin levels (Acrp30) were measured in 50 μL of serum using the LEGEND MAX^™^ Human Adiponectin kit (Biolegend, USA), following the manufacturer’s directions. The samples were analyzed using the Cytation Reader and Gen 5 software (Biotek, USA).

### Gene expression

Expression levels of SIRT1 and PGC-1α genes were determined using the housekeeping gene-independent ACTB (normalizer) by Droplet Digital polymerase chain reaction (ddPCR) using the ddPCR Supermix for gene expression (Bio-Rad). PCR was completed in a T100 thermal cycler and analyzed with a QX200 ddPCR (Bio-Rad). We first isolated total RNA from the globular fraction samples using the TRIzol^®^ method, and we proceeded to generate the cDNA using the RevertAid First-Strand cDNA Synthesis Kit (Thermo Scientific). ddPCR reactions were carried out in triplicate and following the manufacturer’s directions, using specific primers to amplify SIRT1 and PGC-1α. PCR reactions were performed in a T100 thermal cycler (Bio-Rad). After PCR samples were analyzed with a QX200 droplet, data analysis was performed with Quanta Soft analysis software (Bio-Rad).

### ChIP-qPCR

The ChIP experiments were performed by isolating packed blood cells using the red blood cell lysis protocol. The anti-H3 (sc-374669X) and anti-PPARα (sc-398394X) mouse monoclonal antibodies were purchased from Santa Cruz Biotechnology. In the ChIP-qPCR analyses, the values from the immunoprecipitated samples were normalized to that from the input DNA. Primer sequences for ADIPOQ and PGC1-α are in the S5 Table.

### Protein SIRT1 expression by flow cytometry

Cells from blood samples were treated with ammonium chloride-based buffer (10X RCB lysis buffer) followed by a series of washes with a phosphate buffer solution (PBS) of pH 7.4. Cells were suspended at a concentration of 1x 10^6^ cells/ml and incubated with a rabbit anti-SIRT1 antibody (Abcam) for 20 min at 4°C, followed by incubation with an anti-rabbit FITC secondary antibody (eBioscience) for 30 min to detect SIRT1-positive cells. After washing with PBS, the cells were resuspended in a solution with 1% p-formaldehyde and stored at 4°C until analysis in the FACSCanto II flow cytometer (BD). The results were expressed as the percentage of positive cells.

### Statistical analysis

The statistical analysis was performed using GraphPad Prism 8.0 software (GraphPad, USA). Data are expressed as the mean ± SD and median ± IQR for data with a parametric and non-parametric distribution, respectively. Each data set was first analyzed for the data distribution using the Kolmogorov–Smirnov test. For clinical data from each study group, one-way ANOVA and Tukey’s post hoc test were performed. For the relative miRNA expression and cytokine/adipokine levels, the non-parametric Kruskal–Wallis and Dunn’s post hoc test were performed. Spearman’s correlation analysis was applied to identify correlations between different variables. We corrected with the Bonferroni test, and p ˂ 0.05 was considered statistically significant.

## Results

### Clinical and anthropometric characteristics

The sample included 80 subjects, of whom 29 were women and 51 were men. This sample is derived from a total of 1117 participants, which accomplished the inclusion criteria and stratified into four categories according to metabolic health and BMI: 739 participants (66.1%) classified as Metabolically Healthy Normal Weight (MHNW), 303 (27.1%) as Metabolically Healthy Overweight/Obese (MHOW), 20 (1.8%) as Metabolically Unhealthy Normal Weight (MUNW) and 55 (5.0%) as Metabolically Unhealthy Overweight/Obese (MUOW). After random selection, only 20 participants from each group were considered for the molecular and analytical tests.

### Anthropometric and cardiometabolic variables according to BMI and metabolic health status stratification

The anthropometric and cardiometabolic characteristics of the participants in each group are indicated in Table 1. The MHNW and MHOW showed a lower SBP than those in the metabolically unhealthy category (p = 0.002, post hoc analysis) and lower insulin levels (p = 0.004, post hoc analysis). Although HOMA-IR values were high in the metabolically unhealthy groups, they were still in the range considered normal for a HOMA-IR score, but these scores have different threshold values depending on the study population. By contrast, glucose, LDL-C, Total Cholesterol, and Uric Acid did not differ between groups. As expected, differences in BMI, weight, HDL-cholesterol, and triglycerides were found, given that those variables or parameters were part of the selection criteria.

**Table 1:** Metabolic and anthropometric parameters.

	MHNW	MHOW	MUNW	MUOW	p
n	20	20	20	20	
Sex (male, female)	M:14	M:11	M:11	M:15	0.9
	F:6	F:9	F:9	F:5	
Age (years)	18.9 [18–22]	18.6 [18–22]	20.5 [18–22]	19 [18–22]	0.33
Weight (Kg)	60.5±7.9	79.72±10.9	61.92±7.9	88.61±14	**˃0.0001** ‡
Height (m)	1.66±0.07	1.68±0.1	1.67±0.08	1.70±0.09	0.39
Systolic Blood Pressure (SBP) [mmHg]	103.5±9.9	110±11.2	102.1±7.9	115.5±11.4	**0.0002** †
BMI	21.9±1.5	28.2±3.1	22.1±1.9	30.5±4.3	**˃0.0001** ‡
Glucose (mg/dL)	84.8±6.3	86.8±9.8	86.10±11.9	89.75±10.4	0.44
Triglycerides (mg/dL)	72.5±46.5	79±49	182±73.2	199±112.5	**˃0.0001** §
HDL-C (mg/dL)	58.4±10.5	49.3±5.3	34.8±3	36.0±3.4	**˃0.0001** §
LDL-C (mg/dL)	85.55±28.7	95.33±22.3	82.40±25.9	97.86±20.5	0.15
Total Cholesterol (mg/dL)	159.5±31.4	161.9±25.1	160.2±27.9	179.4±27	0.08
Uric Acid (mg/dL)	5.3±1.2	5.9±1.8	6.2±1.7	6.7±1.6	0.06
Insulin (mU/L)	2.85±1.1	3.05±1.6	4.76±2.4	6.49±3.1	**0.004** †
HOMA-IR	0.72±0.3	0.85±0.2	1.32±0.5	1.55±0.8	**0.02** *

MHNW: Normoweight Metabolically Healthy, MUNW: Normoweight Metabolically Unhealthy, MHOW: Overweight/obese Metabolically Healthy, MUOW: Overweight/obese Metabolically Unhealthy, BMI: Body Mass Index, LDL-c: Low-Density Lipoprotein Cholesterol, HDL-c: High-Density Lipoprotein Cholesterol,

*p < .05 compared Metabolic Healthy (MH) vs. Metabolic Unhealthy (MU) groups,

^§^p < 0.0001 compared MHNW vs. MUNW and MUOW and MHOW vs. MUNW and MUOW,

^‡^p < 0.0001 compared MHNW vs. MHOW and MUOW;

^†^p < .01 compared MH vs. MU groups. Multiple group comparisons were analyzed with one-way ANOVA, post-hoc analysis, and unpaired t-test comparing two groups. A chi-squared test was performed for categorical variables (sex).

We analyzed the impact of BMI and metabolic health (MetH) with family predisposition to cardiovascular diseases and type two diabetes mellitus (T2D), sex, levels of Cholesterol, HDL-cholesterol, and uric acid. Interestingly, we found that the MetH has a close relationship with the concentration of uric acid (the uric acid level is related to the role of ROS in healthy young individuals [14] and BMI with a family predisposition to T2D (S1 and S2 Tables).

### Adipokine and cytokine levels

The serum concentrations of adipokines and pro-inflammatory cytokines were evaluated to explain the relationship between several biochemical markers and metabolic health (Table 2). We did not observe any differences between groups related to C-peptide, FGF-23, ghrelin, peptide YY, and IL-10. All our groups showed higher adiponectin levels (p = 0.03), and MHNW and MHOW individuals had higher resistin levels than MUOW and MUNW (p = 0.02, post hoc analysis and S1 Fig). In the case of leptin, we found higher levels in all metabolically unhealthy or overweight groups compared to the MHNW group (Table 2 and S1 Fig). In cytokine serum concentrations, we found differences in the pro-inflammatory cytokines IL-1β, IL-8, and IL-17A. Compared to MUOW, the MHNW group had lower levels of IL-1β (Table 2, p = 0.03 and S1 Fig) and IL-8 (Table 2, p = 0.02 and S1 Fig). Moreover, the MHNW group had lower levels of IL-17A (Table 2, p = 0.006) than MHOW and MUOW, but no difference was detected from the MUNW group (S1 Fig).

**Table 2:** Adipokines and cytokines.

Adipokines (μg/mL)	MHNW	MHOW	MUNW	MUOW	p
**C-peptide**	1.5 ± 0.4	1.55 ± 0.3	1.61 ± 0.5	1.57 ± 0.4	0.72
**FGF-23**	1.0 ± 0.3	0.63 ± 0.5	1.13 ± 0.45	1.16 ± 0.7	0.31
**Ghrelin**	6.4 ± 1.6	7.2 ± 1.7	5.9 ± 2	6.6 ± 2.3	0.52
**Leptin**	3.8 ± 2.4	7 ± 2.4	6.2 ± 2.1	6.7 ± 3.5	**0.03** *
**Peptide YY**	0.23 ± 0.03	0.23 ± 0.07	0.26 ± 0.05	0.25 ± 0.04	0.2
**Resistin**	1.7 ± 0.4	1.6. ± 0.4	1.25 ± 0.4	1.2 ± 0.5	**0.002** †
**Adiponectin (mg/mL)**	13.17 ± 3.5	13.88 ± 2.6	14.51 ± 4.2	11.01 ± 4.9	**0.03** §
**Cytokines (pg/mL)**					
**IL-1β**	230.3 ± 82.1	291.1 ± 86.4	281.8 ± 114.2	320.5 ± 100.9	**0.03** ‡
**IL-8**	473.3 ± 167.5	548.2 ± 147.8	563.3 ± 175.4	606.5 ± 186.5	**0.02** ‡
**IL-10**	188.4 ± 107.7	211.9 ± 114.8	181.1 ± 132.1	151.8 ± 120.5	0.18
**IL-17A**	283.8 ± 78.7	414.1 ± 159.8	351.0 ± 100.8	421.9 ± 164.7	**0.006** ‡*

*p < .05 compared MHNW vs. MHOW, MUNW and MUOW

^‡^p < .05 compared MHNW vs. MUOW,

^†^p < .01 compared MHNW and MHOW vs. MUOW,

^†^*p<0.001 compared MHNW vs. MHOW and MUOW,

^§^p < .05 Difference between all study groups, analyzed with ANOVA test with post-hoc and unpaired t-test comparing two groups.

### Circulating expression of miRNAs

**Fig 2:**
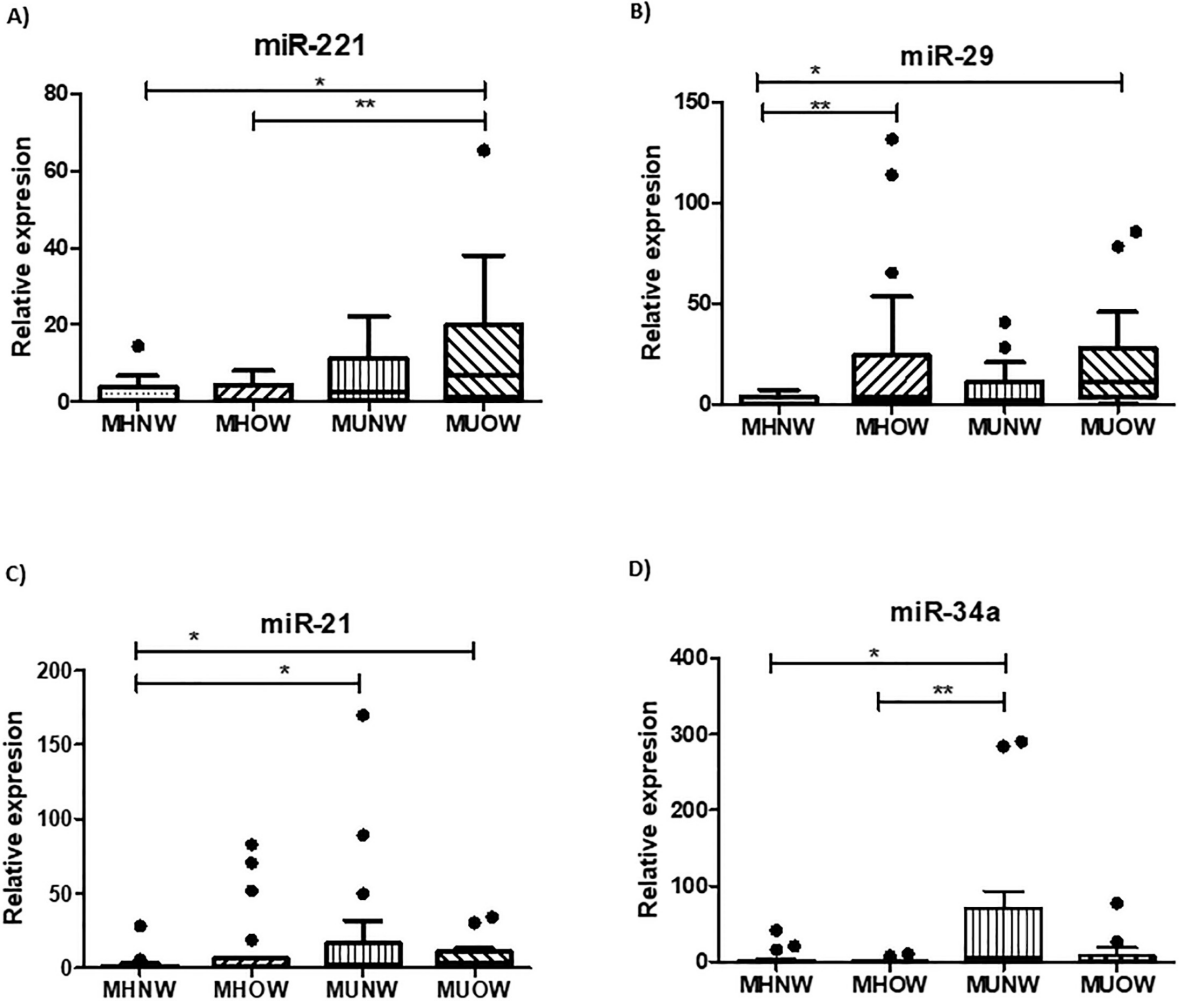
Circulating relative miRNA expression according to body mass index and metabolic health status. Fold change of A) miR-221, B) miR-29a, C) mR-21 and D) miR-34a. The boxes represent medians and interquartile ranges. Data were analyzed using the Kruskal–Wallis and Dunn’s post hoc tests when significant values were obtained (*p < 0.05).

The relative expression of circulating miRNAs miR-221, miR-29a, miR-21, and miR-34a were analyzed, considering participants included in the metabolically healthy and normal-weight group (MHNW) as references. The MHNW group showed lower expression levels of miR-221, miR-29, and miR-21, but not miR-34a, compared with the MUOW group. We observed a significantly higher expression of miR-221 in the metabolically unhealthy groups; the MUOW group showed a higher expression level of miR-221 compared with the MHNW and MHOW groups, suggesting that miR-221 increased gradually based on the metabolic health status and BMI (Fig 2A). A similarly higher expression was observed for miR-29a, but in this case, we detected high expression in both groups of overweight (MHOW and MUOW) participants compared with the reference group (MHNW) (Fig 2B), suggesting an association with BMI but not with metabolic health. Furthermore, for miR-21, we observed a higher expression in the MUNW and MUOW groups compared to the MHNW group; this slight increase in the metabolically unhealthy groups suggested that this miRNA is more associated with metabolic health than with BMI (Fig 2C). The expression of miR-34a showed interesting behavior since its expression was high only in the MUNW compared with the reference MHNW and MHOW groups (Fig 2D).

### Association of BMI and metabolic health with miRNA expression

Due to significant differences in different parameters among the studied groups, a global PCA analysis was performed considering all parameters acquired for each volunteer in our study groups to search for a parameter to cluster our dataset in our study population. In our first approach, the global PCA analysis did not explain much of the variation in the data, indicating a lack of predictability for our study groups. Subsequently, we decided to take a different approach, in which we tested the predictability based on the BMI, stratifying the data according to BMI (normoweight, overweight and obese). We found that PCA1 explained ~12.5% of the variation in our data, observing some separation, but the signal was too weak to claim that our dataset is driven by BMI, which agrees with our proposal of the significant relevance of metabolic health together with BMI (S2 Fig).

**Fig 3:**
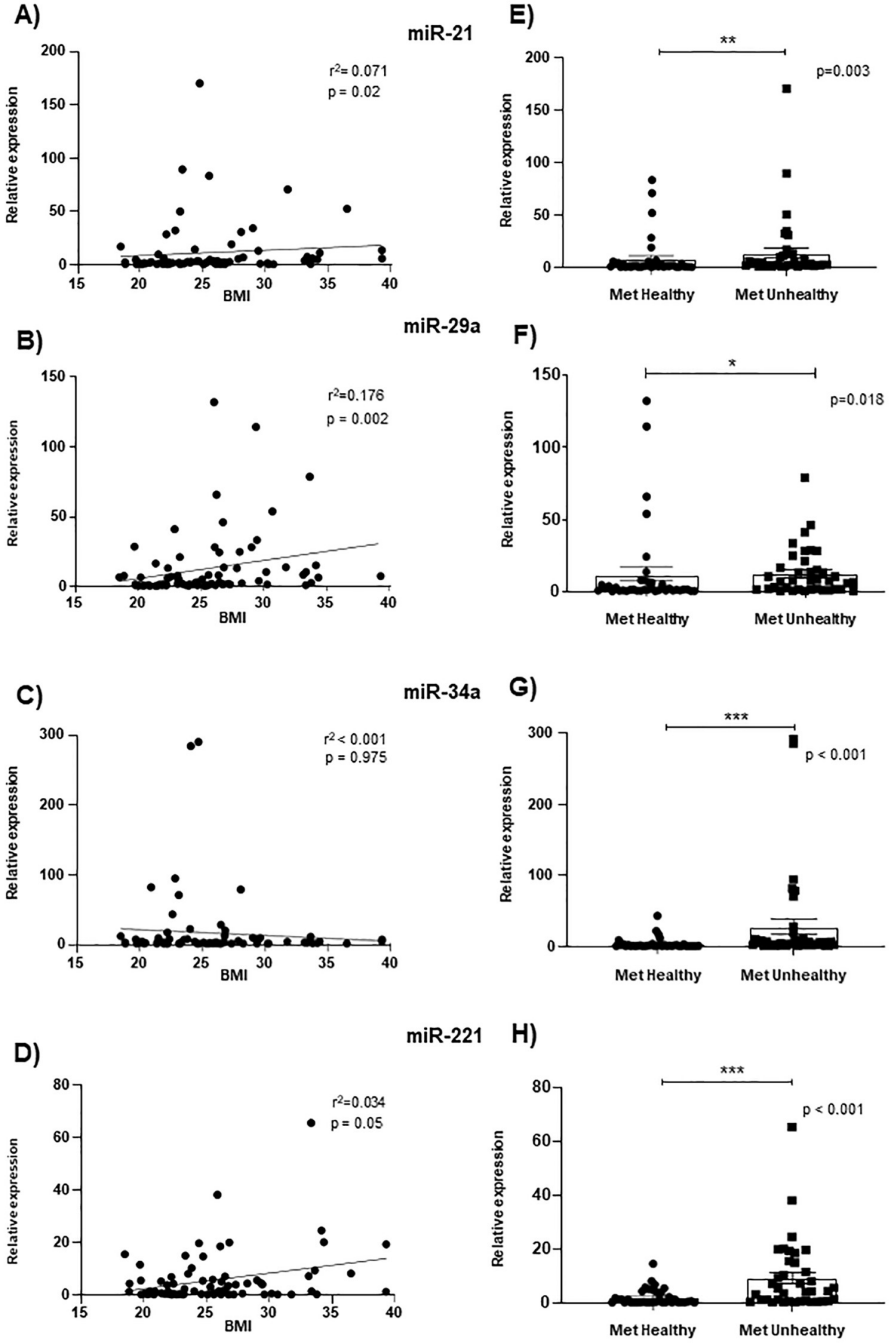
Association between circulating miRNA expression, body mass index, and metabolic health groups. Analysis of circulating A) miR‐21, B) miR-29a, C) miR-34a, and D) miR-221 expression levels associated with BMI and E) miR‐21, F) miR-29a, G) miR-34a and H) miR-221 grouped by metabolic health status. Data was analyzed by Spearman′s correlation test and Mann-Whitney U test; a significant value (p < 0.05) was observed.

Then, we determined the association between BMI and the expression of each circulating miRNA (Fig 3A–3D). In addition, a comparative analysis is shown between metabolically healthy and unhealthy groups with the relative expression of the circulating miRNAs analyzed (Fig 3E–3H). We found that miR-21 and miR-29 were weakly associated with BMI, and no association with miR-34a and miR-221 was found (Fig 3A–3D).

However, when only metabolic status with relative miRNA expression was considered, the statistical analysis showed that all the evaluated miRNAs presented a significantly higher expression in the metabolically unhealthy group than in the metabolically healthy group (Fig 3E–3H). In addition, for circulating miR-29a expression levels, we found higher levels in the obese group (MUOW and MHOW) compared to normoweight group (MHNW) independently if they showed metabolic unhealthy or healthy status in the men group. In contrast, the women showed higher levels of this miR-29 in the MUNW vs. MHNW (S3 Fig). For miR-21 levels, we only found a difference between MUOW vs MHNW in men (S3 Fig). There was no correlation between the circulating expression levels of miR-34a and miR-221 when it is considered only metabolic health status in women or men. Nevertheless, when the groups were stratified based on BMI and metabolic status, differences were detected for miR-34a and miR-221 in men (S4 Fig). In light of these miRNA results, we were interested in verifying if changes had been observed in the expression profiles of miRNA targets in published GEO-based gene expression data sets as proposed by this study. However, it was essential to consider one or two targets for each miRNA since this depends on the expression level of each target and the tissue where these are expressed. Therefore, we used a well-known and comprehensive dataset, the Genotype-Tissue Expression (GTEx v8) project, which is a public resource to study tissue-specific gene expression and regulation (https://gtexportal.org/home/) [32]. First, we found 1,637 miRNAs from 57 healthy tissue types (S5 Fig).

Our analysis, however, supports that miR-34a and miR-221 are upregulated in adipose tissues. Unfortunately, we did not find data for miR-21 or miR-29a in this dataset. Therefore, the expression patterns of miR-34a and miR-221 were studied in key metabolic tissues (S6 Fig). These two miRNAs are primarily overexpressed in adipose tissue, miR-34a being similarly highly expressed in adipose tissue in both subcutaneous and visceral tissue types, as well as being relatively highly expressed in the kidney medulla, which could explain some dysregulation in uric acid. In the case of miR-221, it is also highly expressed in adipose tissue but predominantly expressed in visceral tissue, which could directly support ours and other works relating the levels of this miR-221 with local inflammation in the visceral adipose tissue and dysregulation of lipid uptake.

### Gene expression analysis

**Fig 4:**
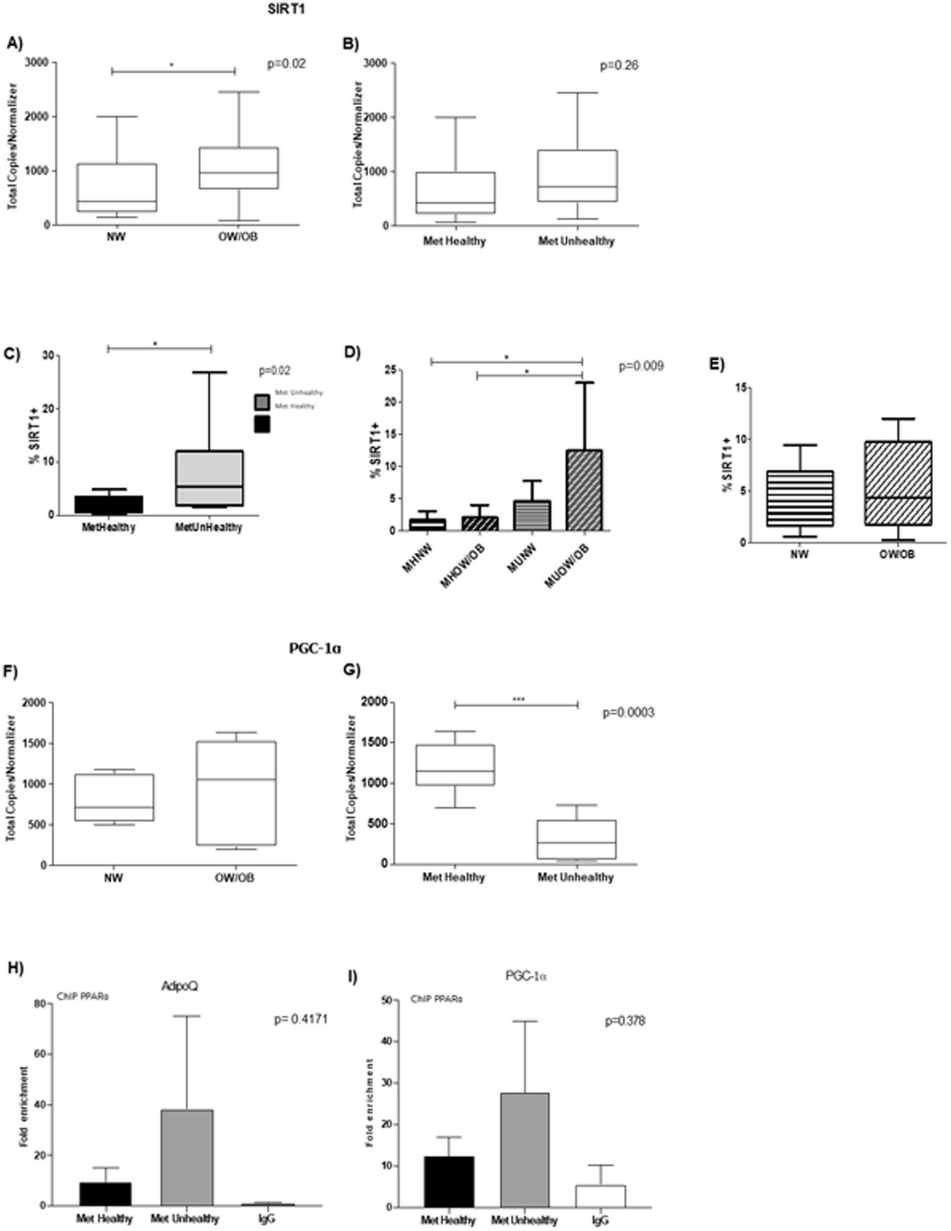
Regulation of gene expression of SIRT1 and PGC1-α. Relative expression of SIRT1 in the globular blood fraction by ddPCR and flow cytometry. SIRT1 expression was analyzed based on BMI (A) and MetH (B). Boxes represent medians and interquartile ranges. Mann–Whitney U test; a significant value *p < 0.05 was observed. SIRT1 protein expression in blood samples and metabolic health (C) or BMI (E). D) Expression of SIRT1 and BMI of metabolically healthy and metabolically unhealthy subjects *p < 0.05. Relative expression of PGC1-α in the globular blood fraction by ddPCR. PGC1-α expression was analyzed based on BMI (F), and G) MetH. Boxes represent medians and interquartile ranges. Mann–Whitney U test; a significant value ***p<0.001 was observed. ChiP-qPCR analysis of PPAR-α with ADIPOQ (H) and PGC1-α (I) promoter regions. Bars represent medians and interquartile ranges, and data were analyzed using the Kruskal–Wallis test and Dunn’s post hoc test when significant values were obtained (*p < 0.05).

SIRT1 is known to regulate PGC-1α and PPAR-α signaling positively and to be regulated by miR-34a; thus, we surveyed SIRT1 and PGC-1α expression in the blood samples and analyzed the data considering BMI and metabolic health status. In the case of SIRT1, we observed significantly higher expression at mRNA (Fig 4A) or protein level (Fig 4C) in the overweight/obese or metabolically unhealthy group than in the normoweight or metabolically healthy group. We also evaluated the correlation between SIRT1 at the protein level with BMI and metabolic health status in the blood samples. A significant difference in SIRT+ cells was detected between the MUOW vs. the MHOW and MHNW subjects (Fig 4D). The latter results were not observed when only the BMI status was evaluated at the protein level (Fig 4E) or considering only metabolic health status at the mRNA level (Fig 4B).

On the other hand, we did not observe any difference in the expression of PGC1-α (Fig 4F) when we performed the analysis based on BMI. However, we detected a significantly higher expression of PGC1-α when we only considered the metabolic health status (Fig 4G). Then, we explored the regulation of SIRT1 and PGC1-α expression through the chromatin accessibility by PPAR-α, a transcription factor that drives sirtuin expression and is considered part of a transcriptional activation loop that regulates the expression of SIRT1-PGC-1α pathways. Therefore, we performed a ChIP-qPCR assay with PPAR-α and evaluated ADIPOQ (adiponectin) and PGC-1α genes related to triglyceride and HDL cholesterol metabolism. We observed similar behaviors for ADIPOQ and PGC-1α, which tended to be more actively transcribed in the metabolically unhealthy group (Fig 4H and 4I).

## Discussion

The evaluation of circulating miRNAs in biological fluids can be used to investigate the biological process of cells and the tissues that synthesize them [33]. In our study, we not only considered BMI as selection criteria to evaluate the metabolic health status of our volunteers but also included biochemical blood data related to lipid metabolism (triglycerides and HDL cholesterol) as metabolic health stratification criteria since both parameters have been shown to have greater predictive power for MetS. In addition, they have been reported as the most prevalent dyslipidaemias among urban Mexican populations [34].

Another approach to identifying the MHOW phenotype includes the homeostasis model assessment of insulin resistance (HOMA-IR) [35]. Interestingly, we observed differences in HOMA-IR and uric acid levels: HOMA-IR in metabolically unhealthy individuals was not above normal values, suggesting that glucose tolerance is already dysregulated because of the MetH classification. Also, we observed a higher level of uric acid in the metabolic unhealthy groups reported before in a similar study, which could indicate intracellular and mitochondrial oxidative stress [21], these being some of the first metabolic features that are altered due to MetH complications. It is essential to mention, although the young life span ranges up to 30 years old, that the age range in our study reflects most changes during the youth-to-adult transition. Further studies related to serum oxidant/antioxidant capacity are important parameters to be included when obesity and metabolic health are involved.

The serum levels of adipokines and cytokines directly related to lipid metabolism, fat storage [25, 36], and inflammation [27, 37] allowed us to evaluate a low-grade inflammation signature given by the metabolic health status and BMI. Specifically, the cytokines IL-1β, IL-8, and IL-17A, which play a central role in controlling a low-grade inflammation status in fat reservoirs, were expressed highly in overweight/obese groups and those with a metabolic unhealthy status. These cytokines represent a pro-inflammatory profile where miR-21, miR-29a, and miR-221 have been reported to control their expression patterns; specifically, miR-29a has been reported to downregulate NF-κB expression in immune cells, upregulating levels of IL-8 and downregulating those of IL-1β [38]. miR-21a and miR-221 have also been shown to downregulate NF-κB, targeting upstream through TNF-α and upregulating IL-1β and promoting a Th17 phenotype in immune cells through IL-17A expression [39, 40]. Likewise, leptin, adiponectin, and resistin were differentially expressed in each metabolic health subgroup. Adiponectin is related to insulin sensitization and is directly associated with lipid and glucose metabolism. Therefore, adiponectin is significant in metabolic health studies as higher levels have been found in heterogeneous populations with metabolically healthy obesity [11, 25, 41], as observed in our MHOW group. For leptin levels, we observed a significantly higher level in the MHOW group, and both MUNW and MUOW groups also showed consistently high leptin expression compared with the MHNW group, which has been more closely related to the known phenotype associated with a high BMI [42]. In addition, the pattern of resistin concentrations in serum is due to their higher levels in both metabolic healthy groups without a direct connection with BMI, suggesting a link between visceral obesity and diabetes in these healthy young individuals [43, 44]. Therefore, early parameters detected in these populations could be relevant to determining their metabolic status [45]. However, a pattern of increased serum resistin levels in metabolic diseases has not previously been reported [46, 47].

We specifically focused on describing the association of the SIRT1/PGC1-α/PPARα signaling pathway with BMI and MetH due to the involvement of fatty acid and glucose metabolism and stress response inflammation [48–50]. It was relevant to the study of gene expression activation by PPAR-α and the potential role of circulating miR-21, miR-29a, miR-34a, and miR-221 to evaluate the main differences in the metabolic phenotypes. These circulating miRNAs play a central role in controlling the establishment and progression of obesity and associated comorbidities like DM2, cardiovascular diseases, and MetS [28, 51, 52]. Specifically, miR-21 negatively regulates the function of PPAR-α [53–55], miR-29a is directly associated with cholesterol metabolism and hepatic nutrient sensing [56–58], miR-34a regulates the function and expression of SIRT1 directly, and miR-221 function has been related to HDL cholesterol metabolism [59, 60]. The increased levels of miR-21 and miR-29a found in the MUOW and MUNW groups could be directly associated with a lower level of PGC-1a mRNA found in the metabolically unhealthy group due to both miRNAs controlling fatty acid metabolism by targeting the PGC-1α/PPAR-α pathway. On the other hand, contrary to our expectations, we observed a higher interaction of PPAR-α with the PGC-1a promoter, which could be a feedback mechanism since levels of fatty acids are higher in the metabolic unhealthy groups, which requires a higher expression of PGC-1α/PPAR-α to metabolize the high levels of circulating fatty acids more quickly [61]. A similar pattern was observed in miR-221, the circulating levels of which gradually increased with unhealthy status, being higher in the MUOW group, which agrees with this unhealthy condition and correlates with the low levels of adiponectin in serum in the MUOW group and a higher interaction of PPAR-α with the ADIPOQ promoter. Therefore, a different mechanism controls adiponectin expression since it is closely related to PPAR-γ [25, 62].

It was not possible to integrate the possible role of circulating miR-34a expression in the intricate mechanism that regulates SIRT1 expression since the expression of this miRNA was higher only in the MUNW group, making it the less prevalent phenotype in our study and the general MetH classification [20, 63]. In addition, we observed a higher SIRT1 mRNA and protein expression in the overweight/obesity group when we considered the BMI classification only, which is the opposite of what we expected since miR-34a targets SIRT1 mRNA directly. However, discordances between levels of SIRT1 protein and mRNA with miR-34a have been previously reported in primary cells or serum samples [24, 60, 64]. Our findings suggest that MHOW, MUNW, and MUOW present several alterations in molecular markers of metabolic alterations and inflammation. Therefore, based on the results of our study, clinical approaches to avoid weight gain and metabolic alterations must not be employed only for individuals with overweight/obesity but should include individuals with an altered metabolic profile. Furthermore, metabolic health interventions regarding lifestyle and weight maintenance should also be directed to individuals with a normal BMI.

## Conclusions

Our study describes for the first time the spectrum of MetH phenotypes and their relationship with BMI classification by identifying distinctive phenotypes among apparently healthy young adults, applying a strict definition consisting of the most common dyslipidaemias in the Mexican population and the absence of comorbidities. Different expression patterns of adipokines, cytokines, and circulating miR-21, miR-29a, miR-34a, and miR-221 have a distinct expression footprint combining the MetH and BMI status. Nonetheless, our findings suggest that beyond simple caloric excess, impaired lipid and glucose metabolism may contribute to the metabolic consequence of obesity later in life. Therefore, additional studies are warranted in a larger sample of individuals to confirm our observations and validate their significance in the different classifications of MetH and anthropometric parameters. The results might have several implications for predicting cardiometabolic diseases, clinical interventions, and drug development. To better understand the metabolic risk parameters with obesity-associated diseases such as hyperglycemia, dyslipidaemias, and hypertension with chronic and metabolic diseases [65].

## Supporting information

S1 Fig Adipokines and cytokines in metabolic health groups. The concentration of relevant adipokines (A) and cytokines (B) in metabolic health groups. Statistical differences between groups were shown as *p≤ 0.05, **p ≤0.01.
(TIF)

S2 Fig Overlayed PCA analysis. BMI (normoweight, overweight and obese).
(TIF)

S3 Fig Circulating expression levels of miR-21 and miR-29a by sex. A) Relative miR‐21 and B) miR-29a expression levels by sex and grouped by metabolic health status and BMI. Data were analyzed by the Kruskal–Wallis test and Mann–Whitney U test; a significant value (p < 0.05) was observed.
(TIF)

S4 Fig Circulating expression levels of miR-34a and miR-221 by sex. A) Relative miR‐34a and B) miR-221 expression levels by sex and grouped by metabolic health status and BMI. Data were analyzed by the Kruskal–Wallis test and Mann–Whitney U test; a significant value (p < 0.05) was observed.
(TIF)

S5 Fig Global expression levels of miRNAs in GTEx RNA-seq. GTEx V8 contains DNA data from 838 post-mortem donors and 17,382 RNA-seq across 54 tissue sites and two cell lines.
(TIF)

S6 Fig miR-34a and miR-221 in GTEx RNA-seq. Normalized expression levels of miR-34a and miR-221 in the GTEx RNA-seq dataset for seven different tissue types.
(TIF)

S1 Table Statistical analysis for binary logistic regression related to metabolic health. (DOCX)

S2 Table Statistical analysis for binary logistic regression related to BMI. (DOCX)

S3 Table RT miRNA primers. (DOCX)

S4 Table miRNAs qPCR primers and probes. (DOCX)

S5 Table ChIP-qPCR primers. (DOCX)

S1 Checklist STROBE statement—Checklist of items that should be included in reports of observational studies. (DOCX)
