# Supplementary figures and images for: miR-21, miR-221, miR-29 and miR-34 are distinguishable molecular features of a metabolically unhealthy phenotype in young adults
Source: PLoS One. 2024 Apr 25;19(4):e0300420. doi: 10.1371/journal.pone.0300420 (PMC11045123; doi:10.1371/journal.pone.0300420)

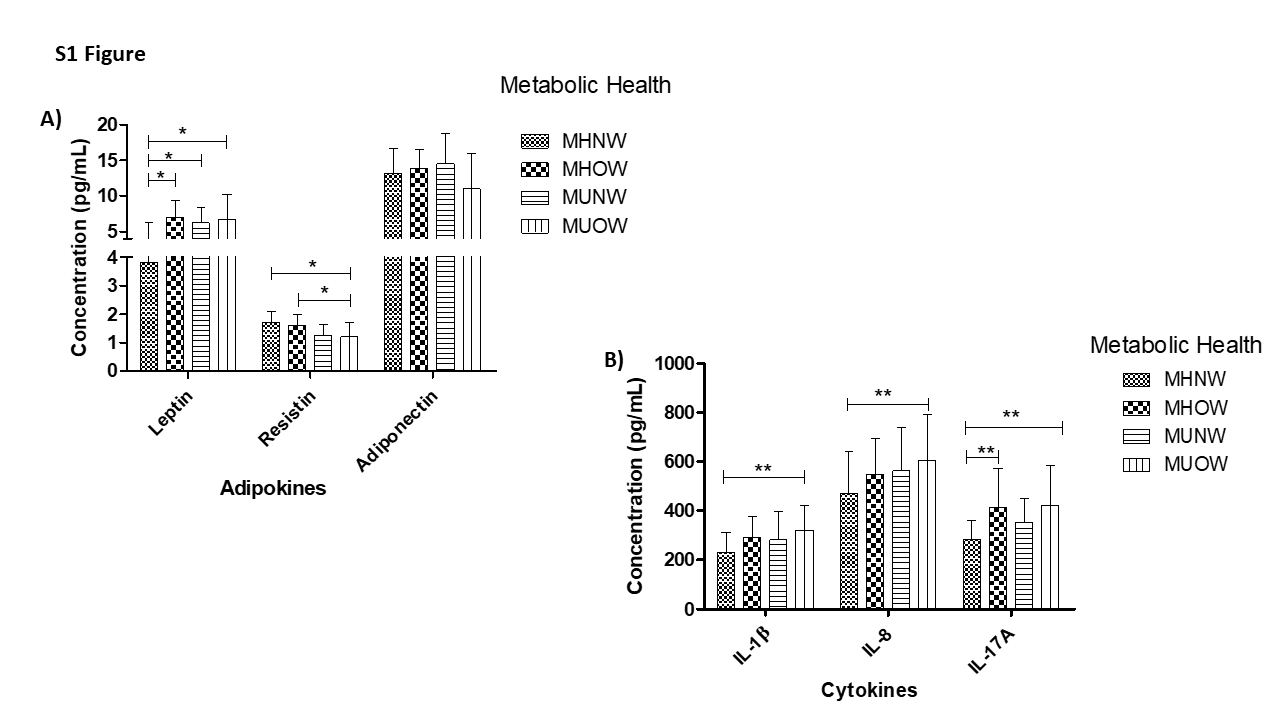

Supplement: S1 Fig — The concentration of relevant adipokines (A) and cytokines (B) in metabolic health groups. Statistical differences between groups were shown as *p≤ 0.05, **p ≤0.01. (TIF) [file pone.0300420.s001.TIF]

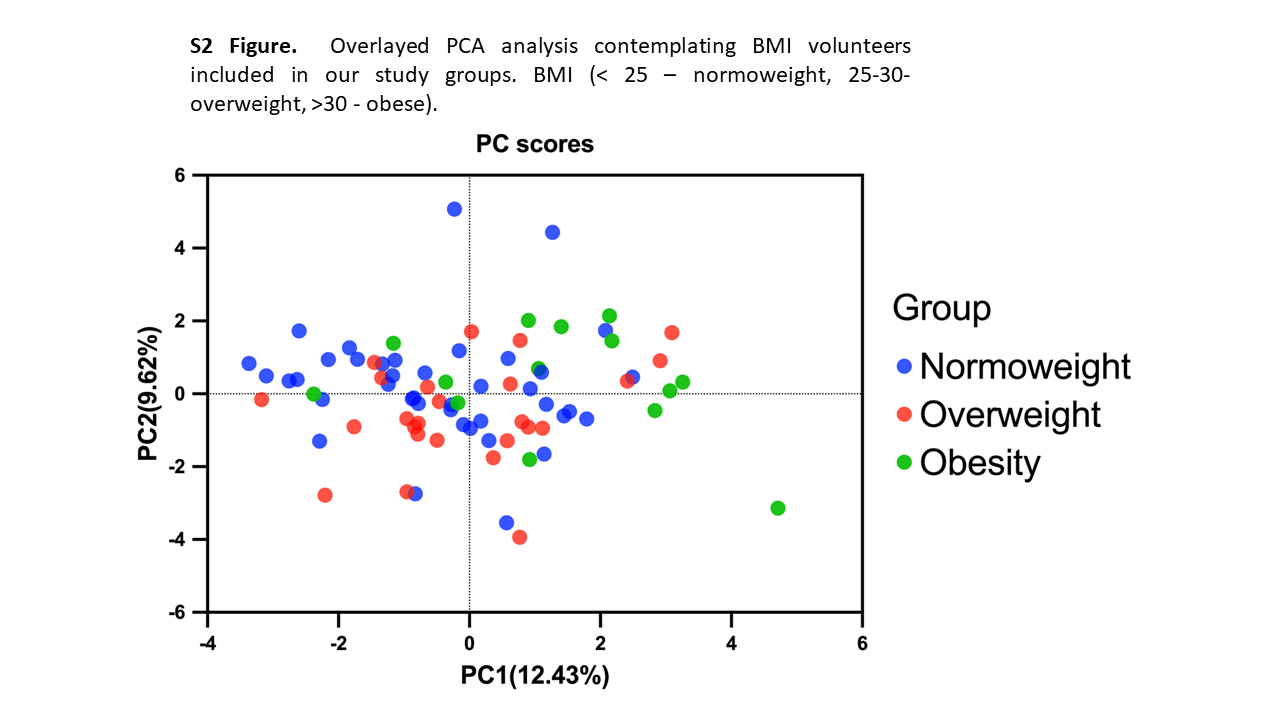

Supplement: S2 Fig — BMI (normoweight, overweight and obese). (TIF) [file pone.0300420.s002.TIF]

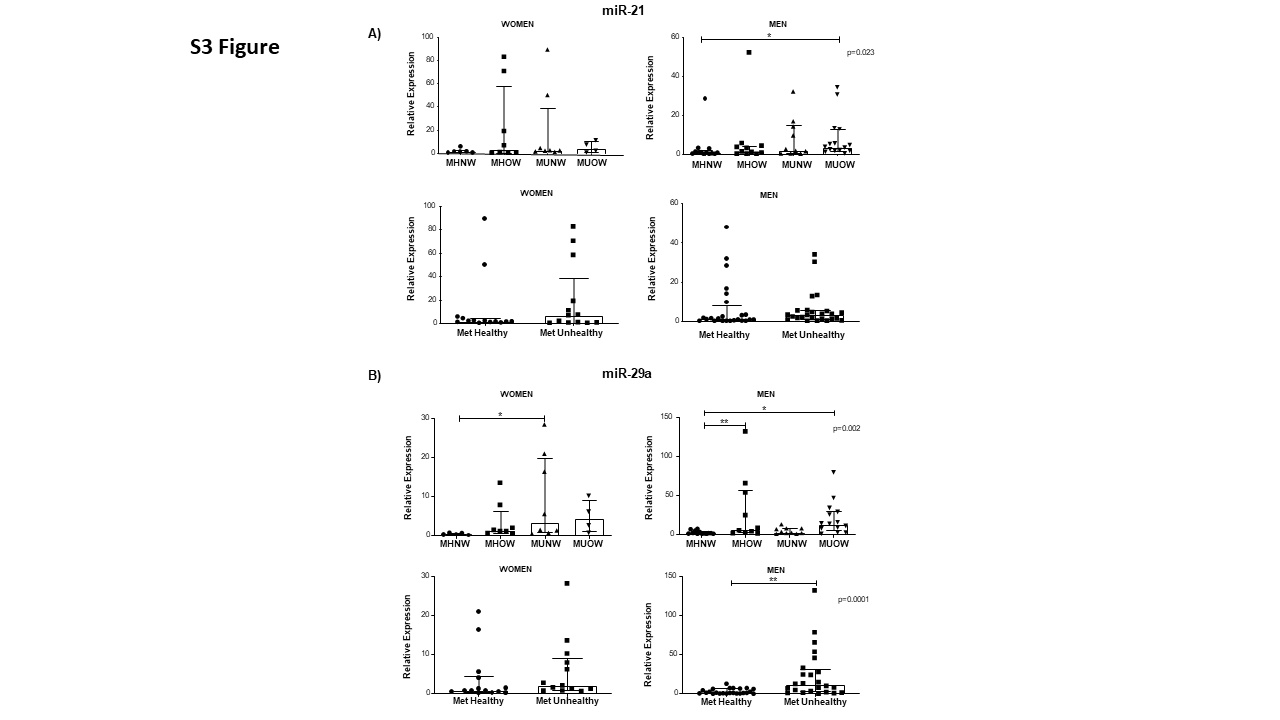

Supplement: S3 Fig — A) Relative miR‐21 and B) miR-29a expression levels by sex and grouped by metabolic health status and BMI. Data were analyzed by the Kruskal–Wallis test and Mann–Whitney U test; a significant value (p < 0.05) was observed. (TIF) [file pone.0300420.s003.TIF]

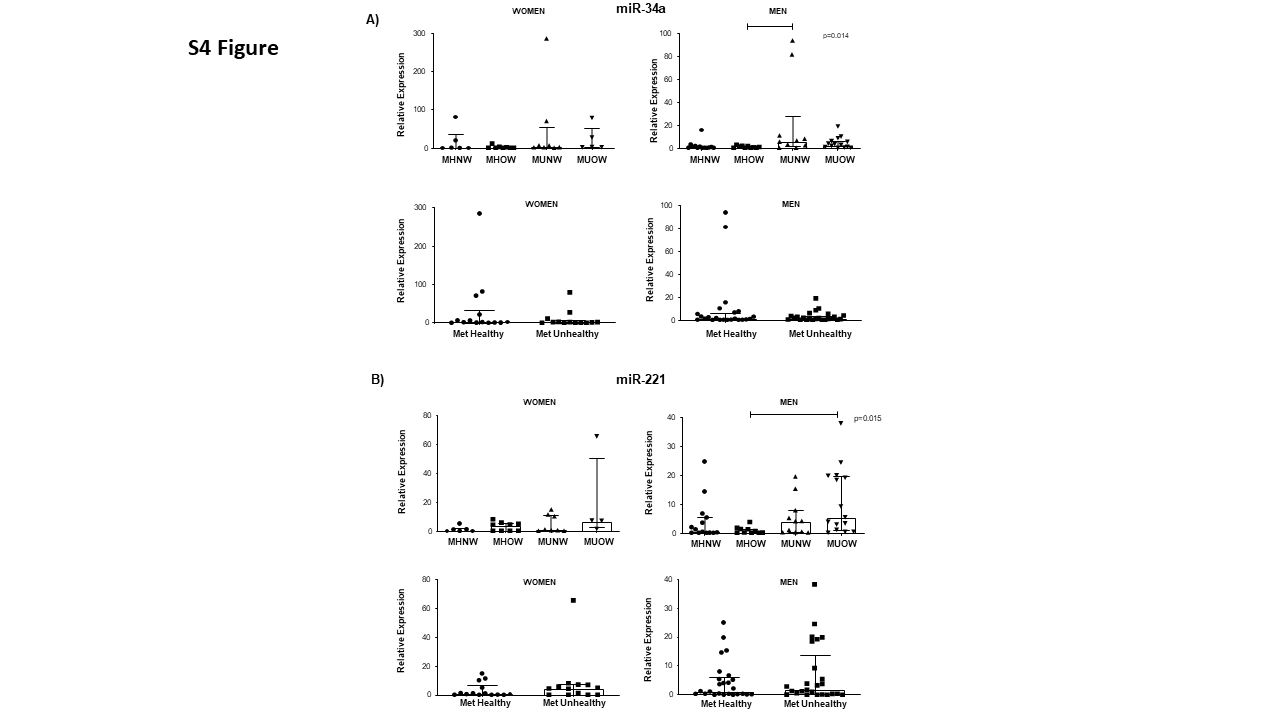

Supplement: S4 Fig — A) Relative miR‐34a and B) miR-221 expression levels by sex and grouped by metabolic health status and BMI. Data were analyzed by the Kruskal–Wallis test and Mann–Whitney U test; a significant value (p < 0.05) was observed. (TIF) [file pone.0300420.s004.TIF]

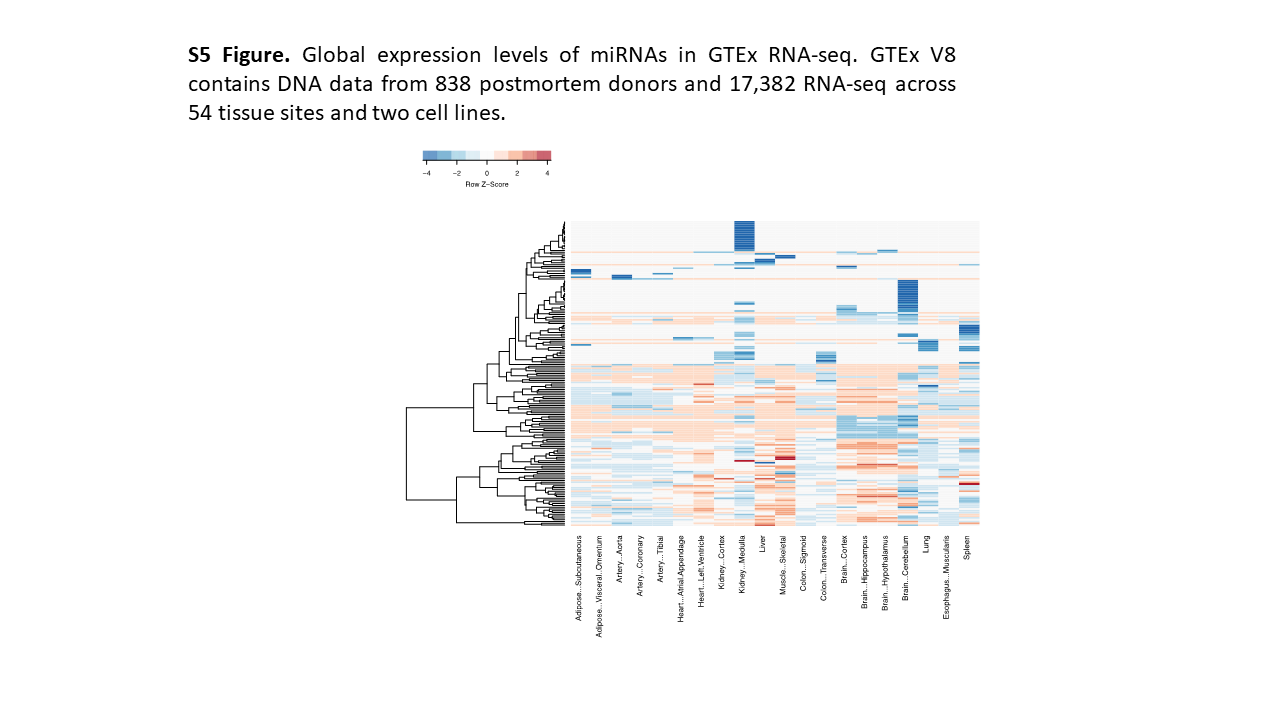

Supplement: S5 Fig — GTEx V8 contains DNA data from 838 post-mortem donors and 17,382 RNA-seq across 54 tissue sites and two cell lines. (TIF) [file pone.0300420.s005.TIF]

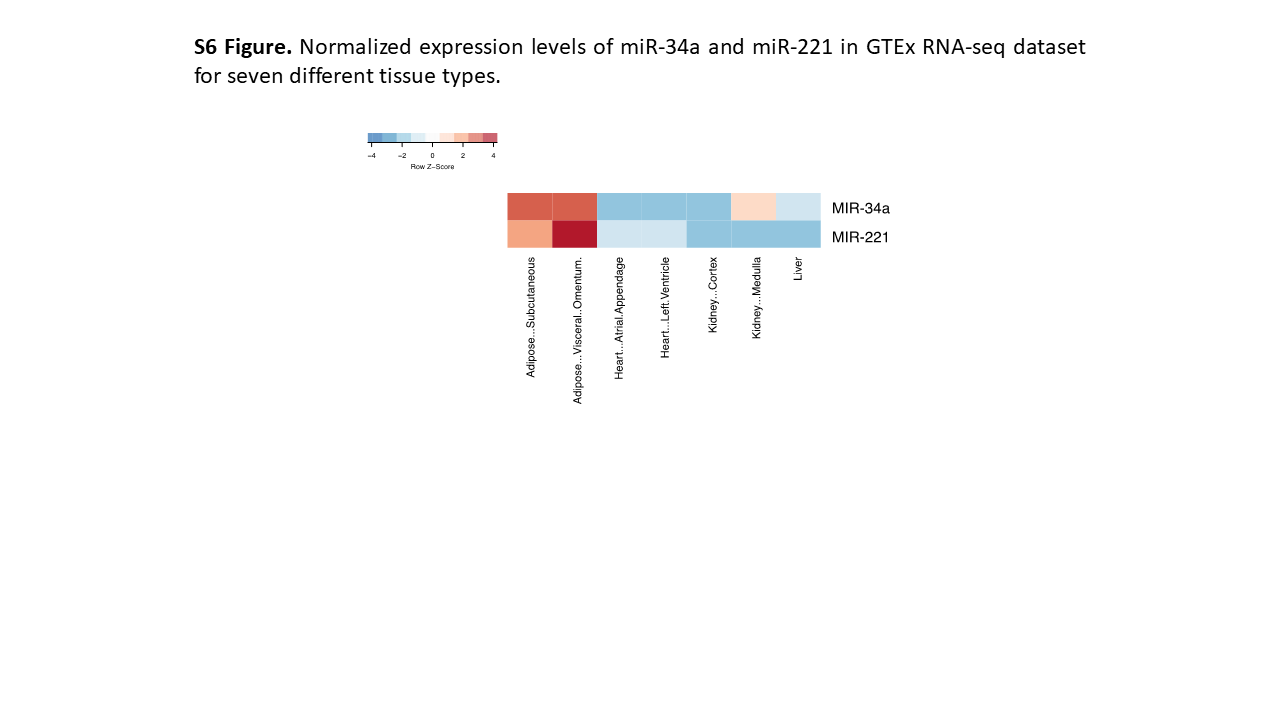

Supplement: S6 Fig — Normalized expression levels of miR-34a and miR-221 in the GTEx RNA-seq dataset for seven different tissue types. (TIF) [file pone.0300420.s006.TIF]
