# Supplementary material for: miR-21, miR-221, miR-29 and miR-34 are distinguishable molecular features of a metabolically unhealthy phenotype in young adults
Source: PLoS One. 2024 Apr 25;19(4):e0300420. doi: 10.1371/journal.pone.0300420 (PMC11045123; doi:10.1371/journal.pone.0300420)
Supplement: S1 Table — (DOCX) [file pone.0300420.s007.docx]

**Supplementary Table 1**

**Statistical analysis for binary logistic regression related to metabolic health**

| Metabolic Health | χ2 (p value) |
| --- | --- |
| DIABETES MELLITUS II | 0.9 |
| CARDIOVASCULAR DISEASE | 0.59 |
| SEX | 0.9 |
| **URIC ACID** | **0.017** |
| CHOLESTEROL | 0.37 |
| LDL-COL | 0.9 |
