# Supplementary material for: miR-21, miR-221, miR-29 and miR-34 are distinguishable molecular features of a metabolically unhealthy phenotype in young adults
Source: PLoS One. 2024 Apr 25;19(4):e0300420. doi: 10.1371/journal.pone.0300420 (PMC11045123; doi:10.1371/journal.pone.0300420)
Supplement: S2 Table — (DOCX) [file pone.0300420.s008.docx]

**Supplementary Table 2**

**Statistical analysis for binary logistic regression related to BMI**

| BMI (kg/m^2^) | χ2 (p value) |
| --- | --- |
| **DIABETES MELLITUS II** | **0.018** |
| CARDIOVASCULAR DISEASE | 0.407 |
| SEX | 0.9 |
| URIC ACID | 0.344 |
| COLESTEROL | 0.76 |
| LDL-COL | 0.9 |
