# Supplementary material for: miR-21, miR-221, miR-29 and miR-34 are distinguishable molecular features of a metabolically unhealthy phenotype in young adults
Source: PLoS One. 2024 Apr 25;19(4):e0300420. doi: 10.1371/journal.pone.0300420 (PMC11045123; doi:10.1371/journal.pone.0300420)
Supplement: S3 Table — (DOCX) [file pone.0300420.s009.docx]

**Supplementary Table 3**

**RT miRNA primers**

| miRNA | RT-stem loop |
| --- | --- |
| hsa-miR-21 | 5’-GTGTCGTCGGGGACCGGGCGAGCTATGGCTAAGGTACCCGGTCCCCGACGACACTCAACT-3’ |
| hsa-miR-34a | 5’-GTTGGCTCTGGTGCAGGGTCCGAGGTATTCGCACCAGAGCCAACACAACC- 3’ |
| hsa-miR-221 | 5’-GTGTCGTCTTGGACAGGGTCCGCGTTGGTCCAGCTAAGGTACCTGTCCAAGACGAAACC-3’ |
| cel-miR-39 | 5’-GTGTCGTCTTGGACAGGGTCCGTTGGTCCAGCTAAGGTACCTGTCCAAGACGACACCAAGCT-3’ |
