# Supplementary material for: miR-21, miR-221, miR-29 and miR-34 are distinguishable molecular features of a metabolically unhealthy phenotype in young adults
Source: PLoS One. 2024 Apr 25;19(4):e0300420. doi: 10.1371/journal.pone.0300420 (PMC11045123; doi:10.1371/journal.pone.0300420)
Supplement: S4 Table — (DOCX) [file pone.0300420.s010.docx]

**Supplementary Table 4**

**miRNAs qPCR primers and probes.**

|  | Forward primer | Reverse Primer | Probe |
| --- | --- | --- | --- |
| hsa-miR-21 | 5’-CTACCGTAGCTTATCAGACTGA-3’ | 5’-CCGGGCGAGCTATGGCT-3’ | 5’-CCGGTCCCCGACGACACTCAACA-TexasRed-3’ |
| hsa-miR-34a | 5’-TCGCGTGGCAGTGTCTTAGCT-3’ | 5’-GTGCAGGGTCCGAGGT-3’ | 5’-CACCAGAGCCAACACAACC-Cy5-3’ |
| hsa-miR-221 | 5’-CGGAGCTACATTGTCTGCTG-3’ | 5’-CAGGGTCCGTTGGTCC-3’ | 5’-CTGTCCAAGACGACACGAAACC-HEX-3’ |
| cel-miR-39 | 5’-CTACACCGGGTGTAAATC-3’ | 5’-CAGGGTCCGTTGGTCC-3’ | 5’-CTGTCCAAGACGACACCAAGCT-FAM-3’ |
