# Supplementary material for: miR-21, miR-221, miR-29 and miR-34 are distinguishable molecular features of a metabolically unhealthy phenotype in young adults
Source: PLoS One. 2024 Apr 25;19(4):e0300420. doi: 10.1371/journal.pone.0300420 (PMC11045123; doi:10.1371/journal.pone.0300420)
Supplement: S5 Table — (DOCX) [file pone.0300420.s011.docx]

**Supplementary Table 5**

**ChIP-qPCR primers**

|  | Forward primer | Reverse Primer |
| --- | --- | --- |
| ADIPOQ | 5’-TCCTAAATCCAGGGTCCAG-3’ | 5’-CACCACAGTCTTGCTCAC-3’ |
| PGC1-α | 5’-TGAGGGCAGAGCCAATGA-3’ | 5’-GCTGAATACAGTTCACCAGCAC-3’ |
| ACTIN | 5’- GCTGTTCCAGGCTCTGTTCC -3’ | 5’-ATGCTCACACGCCACAACATGC-3’ |
