# Supplementary material for: miR-21, miR-221, miR-29 and miR-34 are distinguishable molecular features of a metabolically unhealthy phenotype in young adults
Source: PLoS One. 2024 Apr 25;19(4):e0300420. doi: 10.1371/journal.pone.0300420 (PMC11045123; doi:10.1371/journal.pone.0300420)
Supplement: S1 Checklist — (DOCX) [file pone.0300420.s012.docx]

STROBE Statement—checklist of items that should be included in reports of observational studies

|  | Item No. | Recommendation | Page  No. | Relevant text from manuscript |
| --- | --- | --- | --- | --- |
| **Title and abstract** | 1 | (*a*) Indicate the study’s design with a commonly used term in the title or the abstract | 2 | We conducted a cross-sectional molecular study on 80 individuals classified into four subgroups according to their metabolic health and BMI |
|  |  | (*b*) Provide in the abstract an informative and balanced summary of what was done and what was found | 2 | We found differences in the SIRT1-PGC1α pathway and circulating miRNA expression: mIR-21 and miR-29a levels were altered by MetH status. There was a direct association of IL-8, IL-17A, leptin and resistin with metabolic unhealthy and OW/OB phenotypes. Our results demonstrate that MetH and BMI have a distinguishable molecular marker signature that can predict the potential to develop major metabolic diseases: miR-221, miR-21, resistin and IL-17A were differently expressed between the groups studied. |
| Introduction | | | |  |
| Background/rationale | 2 | Explain the scientific background and rationale for the investigation being reported | 3-5 | It was included in the introduction section. |
| Objectives | 3 | State specific objectives, including any prespecified hypotheses | 5,6 | We aimed to categorize the healthy and unhealthy young adult population and perform detailed metabolic phenotyping, including the molecular marker signature (cardiometabolic, adiposity, inflammation, and epigenetic markers) in the blood to provide a better explanation of the function of adipose tissue, its effects at the systemic level and the prevention of obesity. In the present study, we hypothesize that metabolic health status shows a distinctive molecular signature (circulating miRNA expression and metabolic/inflammation biomarkers) that is not observed when considering BMI alone as the selection criterion and will allow better differentiation of the phenotypes of metabolic health. |
| Methods | | | |  |
| Study design | 4 | Present key elements of study design early in the paper | Fig 1 | Figure 1. Overall study design flowchart. |
| Setting | 5 | Describe the setting, locations, and relevant dates, including periods of recruitment, exposure, follow-up, and data collection | 5 | A cross-sectional study was conducted on 1469 young adults aged 18–24 years, applicants to a public university in Mexico recruited in the Health Services Department during the standard admission process in July 2016. |
| Participants | 6 | (*a*) *Cohort study*—Give the eligibility criteria, and the sources and methods of selection of participants. Describe methods of follow-up  *Case-control study*—Give the eligibility criteria, and the sources and methods of case ascertainment and control selection. Give the rationale for the choice of cases and controls  *Cross-sectional study*—Give the eligibility criteria, and the sources and methods of selection of participants | 5-6 | The health screen consisted of 1) anthropometric measurements, including height and weight, 2) a blood draw following an overnight fast for biological markers and 3) blood pressure measurement. Students with a previous diagnosis of diabetes, cardiovascular, autoimmune, or renal disease, or cancer and those under treatment with anti-inflammatories and steroids at the time of recruitment were excluded.  **Stratification according to BMI and metabolic health status**. |
|  |  | (*b*) *Cohort study*—For matched studies, give matching criteria and number of exposed and unexposed  *Case-control study*—For matched studies, give matching criteria and the number of controls per case |  | NA |
| Variables | 7 | Clearly define all outcomes, exposures, predictors, potential confounders, and effect modifiers. Give diagnostic criteria, if applicable | 5 | A previous diagnosis of diabetes, cardiovascular, autoimmune, or renal disease, or cancer and those under treatment with anti-inflammatories and steroids at the time of recruitment were excluded. |
| Data sources/ measurement | 8* | For each variable of interest, give sources of data and details of methods of assessment (measurement). Describe comparability of assessment methods if there is more than one group | 7,8 | It was described in the material and methods section. |
| Bias | 9 | Describe any efforts to address potential sources of bias | 6 | It was applied a randomized sampling method to select the individual in each group. |
| Study size | 10 | Explain how the study size was arrived at | 6 | 80 individuals were selected by randomized sampling using the Research Randomizer (Version 4.0). |

Continued on next page

| Quantitative variables | | 11 | | Explain how quantitative variables were handled in the analyses. If applicable, describe which groupings were chosen and why | 8, 9, 10 | | Data are expressed as the mean ± SD and median ± IQR for data with a parametric and non-parametric distribution, respectively. In addition, in results section it was included a table with those quantitative variables evaluated. |
| --- | --- | --- | --- | --- | --- | --- | --- |
| Statistical methods | | 12 | | (*a*) Describe all statistical methods, including those used to control for confounding | 8-9 | | The statistical analysis was performed using GraphPad Prism 8.0 software (GraphPad, USA). Each data set was first analysed for the data distribution using the Kolmogorov–Smirnov test. For clinical data from each study group, one-way ANOVA and Tukey’s post hoc test were performed. For the relative miRNA expression and cytokine/adipokine levels, the non-parametric Kruskal–Wallis and Dunn’s post hoc test were performed. Spearman’s correlation analysis was applied to identify correlations between different variables. We corrected with the Bonferroni test, and p ˂ 0.05 was considered statistically significant. |
|  |  |  |  | (*b*) Describe any methods used to examine subgroups and interactions |  | | In Table 1 |
|  |  |  |  | (*c*) Explain how missing data were addressed |  | | NA |
|  |  |  |  | (*d*) *Cohort study*—If applicable, explain how loss to follow-up was addressed  *Case-control study*—If applicable, explain how matching of cases and controls was addressed  *Cross-sectional study*—If applicable, describe analytical methods taking account of sampling strategy. |  | | NA  Material and Methods (page 7) and results sections (page 9) described the sampling strategy. |
|  |  |  |  | (*e*) Describe any sensitivity analyses | NA | |  |
| Results | | | | | | | |
| Participants | | 13* | | (a) Report numbers of individuals at each stage of study—eg numbers potentially eligible, examined for eligibility, confirmed eligible, included in the study, completing follow-up, and analysed | 9 | | The sample included 80 subjects, of whom 29 were women and 51 were men. After random selection, only 20 participants from each group were considered for the molecular and analytical tests. |
|  |  |  |  | (b) Give reasons for non-participation at each stage |  | | NA |
|  |  |  |  | (c) Consider use of a flow diagram | 7 | | Figure 1. Overall study design flowchart. |
| Descriptive data | | 14* | | (a) Give characteristics of study participants (eg demographic, clinical, social) and information on exposures and potential confounders | 9-10 | | The anthropometric and cardiometabolic characteristics of the participants in each group are indicated in Table 1 |
|  |  |  |  | (b) Indicate number of participants with missing data for each variable of interest |  | | NA |
|  |  |  |  | (c) *Cohort study*—Summarise follow-up time (eg, average and total amount) |  | | NA |
| Outcome data | | 15* | | *Cohort study*—Report numbers of outcome events or summary measures over time |  | | NA |
|  |  |  |  | *Case-control study—*Report numbers in each exposure category, or summary measures of exposure |  | | NA |
|  |  |  |  | *Cross-sectional study—*Report numbers of outcome events or summary measures |  | | *In table 1 and table 2 are described* |
| Main results | | 16 | | (*a*) Give unadjusted estimates and, if applicable, confounder-adjusted estimates and their precision (eg, 95% confidence interval). Make clear which confounders were adjusted for and why they were included |  | | *NA* |
|  |  |  |  | (*b*) Report category boundaries when continuous variables were categorized |  | | NA |
|  |  |  |  | (*c*) If relevant, consider translating estimates of relative risk into absolute risk for a meaningful time period |  | | *NA* |
| Continued on next page Other analyses | 17 | | Report other analyses done—eg analyses of subgroups and interactions, and sensitivity analyses | |  | NA | |
| Discussion | | | | | | | |
| Key results | 18 | | Summarise key results with reference to study objectives | | 18 | Our findings suggest that MHOW, MUNW and MUOW present several alterations in molecular markers of metabolic alterations and inflammation. Therefore, based on the results of our study, clinical approaches to avoid weight gain and metabolic alterations must not be employed only for individuals with overweight/obesity but should include individuals with an altered metabolic profile. Furthermore, metabolic health interventions regarding lifestyle and weight maintenance should also be directed to individuals with a normal BMI. | |
| Limitations | 19 | | Discuss limitations of the study, taking into account sources of potential bias or imprecision. Discuss both direction and magnitude of any potential bias | | 18 | Additional studies are warranted in a larger sample of individuals to confirm our observations and validate their significance in the different classifications of MetH and anthropometric parameters. | |
| Interpretation | 20 | | Give a cautious overall interpretation of results considering objectives, limitations, multiplicity of analyses, results from similar studies, and other relevant evidence | | 18 | Our study describes for the first time the spectrum of MetH phenotypes and its relationship with BMI classification by identifying distinctive phenotypes among apparently healthy young adults, applying a strict definition consisting of the most common dyslipidaemias in the Mexican population and the absence of comorbidities. Different expression patterns of adipokines, cytokines and circulating miR-21, miR-29a, miR-34a and miR-221 have a distinct expression footprint combining the MetH and BMI status. Nonetheless, our findings suggest that beyond simple caloric excess, impaired lipid and glucose metabolism may contribute to the metabolic consequence of obesity later in life. | |
| Generalisability | 21 | | Discuss the generalisability (external validity) of the study results | | 15-18 | In discussion section was included. | |
| Other information | | |  | | | | |
| Funding | 22 | | Give the source of funding and the role of the funders for the present study and, if applicable, for the original study on which the present article is based | | 19 | Méndez-Mancilla A was a recipient of a scholarship (412653) from CONACYT, México. | |

*Give information separately for cases and controls in case-control studies and, if applicable, for exposed and unexposed groups in cohort and cross-sectional studies.

**Note:** An Explanation and Elaboration article discusses each checklist item and gives methodological background and published examples of transparent reporting. The STROBE checklist is best used in conjunction with this article (freely available on the Web sites of PLoS Medicine at http://www.plosmedicine.org/, Annals of Internal Medicine at http://www.annals.org/, and Epidemiology at http://www.epidem.com/). Information on the STROBE Initiative is available at www.strobe-statement.org.
